# How does exposure to masked individuals affect White Americans' attitudes toward Asian American and Pacific Islanders?

**DOI:** 10.1111/spc3.12819

**Published:** 2023-06-22

**Authors:** Kimberly Rios, Carolyn T. Pham, Zhen Hadassah Cheng, Bobby K. Cheon

**Affiliations:** 1 Department of Psychology, Ohio University, Athens, Ohio, USA; 2 Department of Psychology, DePaul University, Chicago, Illinois, USA; 3 Oregon Health and Sciences University, Portland, Oregon, USA; 4 National Institute of Child Health and Human Development, Bethesda, Maryland, USA

**Keywords:** attitudes toward AAPIs, COVID-19 attitudes, face masks, realistic threat, symbolic threat

## Abstract

Across three studies, conducted between Spring 2020 and Spring 2021, we tested whether exposure to an Asian American/Pacific Islander (AAPI) target person wearing a face mask would increase or decrease White Americans' perceived threat from and positivity toward AAPI individuals. Although results varied by study, a single-paper meta-analysis revealed that the masked (compared to unmasked) AAPI target resulted in greater positivity toward AAPIs, due to reduced perceptions of both symbolic threat to group values and realistic threat to group health. Positivity toward AAPIs did not reliably differ after exposure to a masked versus unmasked White target. Implications for interventions that encourage COVID-safe behaviors and combat anti-AAPI attitudes are discussed.

## 1 | INTRODUCTION

Before COVID-19, the wearing of face masks was relatively uncommon in the US, despite being prevalent in many East Asian countries (Leung, 2020; Wong, 2020). As COVID-19 cases rose, however, so did mask-wearing, especially following US government recommendations (Goldberg et al., 2020). Unfortunately, increases in COVID-19 cases also coincided with increased negativity toward Asian American and Pacific Islander (AAPI) individuals (Dhanani & Franz, 2020; Mandalaywala et al., 2023; Nguyen et al., 2020; Tessler et al., 2020). Although research suggests that AAPIs are sometimes apprehensive about wearing masks in public due to not wanting to be seen as strange or as carriers of the virus (Kahn & Money, 2022), less is known about how AAPIs who wear (vs. do not wear) masks are perceived. In our research, we examine how masks impact White Americans' perceptions of threat from and attitudes toward AAPIs as a group.

Intergroup Threat Theory offers a lens through which to uncover specific sources of anti-AAPI attitudes during COVID-19. According to this theory, perceived realistic threat (i.e., a tangible attack on the ingroup's physical, economic, or material well-being) and symbolic threat (i.e., an attack on the ingroup's sociocultural identity, including values, culture, and way of life) can independently predict outgroup prejudice (Rios et al., 2018; Stephan et al., 2015). Further, realistic threat encompasses threats to the ingroup's power, status, and economic resources (*realistic status threat*) as well as threats to the ingroup's health and physical safety (*realistic health threat*) (Abrams et al., 2017; Rios et al., 2018). A recent US study found that COVID-19 itself is associated with perceptions of both realistic and symbolic threat (Kachanoff et al., 2021). Similarly, AAPIs may also be viewed as either a realistic or symbolic threat within the context of the pandemic. For example, AAPIs may elicit perceived realistic threat if they are seen as likely to carry the virus (i.e., realistic health threat) or to take coveted jobs during economic downturns (i.e., realistic status threat) (Maddux et al., 2008; Morrison & Ybarra, 2008; Tessler et al., 2020), as well as perceived symbolic threat if they are seen as “perpetually foreign” and thus different from most other Americans (Cheryan & Monin, 2005; Devos & Banaji, 2005; Zou & Cheryan, 2017).

One factor that may influence perceived threat from AAPIs during COVID-19 is the increased wearing of face masks. Some research has identified predictors of attitudes toward mask-wearing. For example, men are more likely than women to view mask-wearing as infringing on their independence (Howard, 2021). Political conservatives feel more comfortable around both AAPI and White individuals who do not wear masks (Boykin et al., 2021), and people who oppose political correctness norms evaluate masks more negatively and are less likely to wear masks (Mackey et al., 2023). However, aside from the study on political conservatives' comfort with mask-wearers (Boykin et al., 2021), little is known about how members of different ethnic groups are perceived when they wear masks.

## 2 | HYPOTHESES

We propose two competing hypotheses for how mask-wearing might affect attitudes toward AAPIs. On one hand, masks could heighten perceived threat from and negativity toward AAPIs. Consistent with research on AAPIs' feelings of anxiety about how they will be viewed while wearing a mask (Kahn & Money, 2022), people may associate AAPIs who wear masks with being foreign (i.e., symbolic threat), being carriers of COVID-19 and hence more likely to spread the virus (i.e., realistic health threat), or both. On the other hand, given that mask-wearing has become more prevalent in the US over time and is even mandated in certain contexts (Friedman, 2020; Goldberg et al., 2020), mask-wearing could be seen as a symbol of *greater* rather than less “American-ness.” As a result, AAPIs who wear masks may be viewed as less of a symbolic threat than those who do not, and hence be evaluated more positively. Additionally, perhaps AAPIs who wear masks are viewed as doing their part to protect others from the virus and its spread, therefore reducing perceived realistic health threat. ^1^

We conducted three separate studies with White American participants but combined them to increase statistical power, due to their nearly identical methodologies (See Supplementary Materials for the results of each individual study.). We restricted our analyses to White Americans in part because of the limited availability of participants of color in our samples, which rendered us underpowered to detect any effects of participant race. Further, the complexity of intra-minority group relations may have made such effects difficult to interpret without a priori predictions (Craig & Richeson, 2016).

Participants in all studies viewed a photograph of either an AAPI or White target person, who was either wearing a mask or no mask. Participants then answered questions about their perceptions of and positivity toward AAPIs generally. We hypothesized that masked AAPI targets who wore masks would elicit either heightened or reduced threat perceptions, compared to unmasked AAPI targets. We did not expect these effects to emerge with masked (vs. unmasked) White targets because we had no reason to anticipate that exposure to such targets would directly affect positivity toward AAPIs. ^2^ Additionally, we hypothesized that among participants exposed to AAPI—but not White—targets, threat perceptions would mediate the relationship between mask condition and positivity toward AAPIs.

## 3 | METHOD

Participants. Seven hundred and thirty-eight US residents participated in exchange for $1.00 on Prolific Academic (Studies 1 and 3), or for partial psychology course credit at the first author's university (Study 2). The data from 47 participants who did not self-identify as White, 33 participants who did not correctly identify the race of the target person, and four participants who took more than an hour to complete the study were omitted from analyses. The final sample consisted of 614 individuals (382 women, 214 men, 3 transgender men, 4 transgender women, 9 gender non-binary/gender-fluid, 2 other gender; *M*_age_ = 30.36, *SD* = 13.46, range = 17–79).

Participants were randomly assigned to view a picture of either an AAPI (*n* = 279) or White (*n* = 335) individual, who was either masked (*n* = 300) or unmasked (*n* = 314).

Procedure and Materials. After providing informed consent, participants read that the purpose of the study was to examine perceptions of different individuals and groups. They then answered a few basic demographic questions. On the following screen, participants viewed a photograph of their assigned target person. The target person was either AAPI or White and was either wearing a plain white cloth mask over their nose and mouth (photoshopped onto the picture) or was not wearing a mask. The gender of the target person always matched the gender of the participant; participants who self-identified as gender non-binary/gender-fluid were randomly assigned to view either a male or female target. The photographs were taken from the Chicago Face Database (Ma et al., 2015), which contains data on independent raters' perceptions of each target person's race, gender, age, attractiveness, and other characteristics (e.g., emotional expressions). All selected target persons were rated as being in their early- to mid-20s and of roughly average physical attractiveness (i.e., just above a 3 out of 5); in addition, raters needed to correctly identify the targets' race at least 95% of the time (See Supplementary Materials for all pictures.).

Participants were asked to imagine that they had seen the target person at a local grocery store (Study 1), local park (Study 2), or busy local street (Study 3). Below the photograph, they responded to several filler items, unrelated to the present research question, about their perceptions of the target person to disguise the purpose of the study.

Participants then completed an 11-item measure of perceived threat from AAPIs (“Asian people” in Studies 1–2, “Asian Americans” in Study 3; see Supplementary Materials). This measure contained three items assessing perceived realistic status threat (e.g., “To what extent do you think that increases in Asian Americans' status will reduce other Americans' status?”; *M* = 1.46, *SD* = 0.95, *α* = 0.92), four items assessing perceived symbolic threat (e.g., “To what extent do you feel that Asian Americans are threatening other Americans' core values?”; *M* = 1.31, *SD* = 0.85, *α* = 0.95), and four items assessing perceived realistic health threat (e.g., “To what extent do you think that Asian Americans are threatening the health of other groups in the US?”; *M* = 1.44, *SD* = 0.99, *α* = 0.97). All items were administered on 7-point scales (1 = *strongly disagree*, 7 = *strongly agree*).

Next, participants completed a four-item measure of overall attitudes toward AAPIs. The first item was a “feeling thermometer” from 0 (*no warmth at all*) to 100 (*complete warmth*), on which participants indicated their feelings of warmth toward Asian people (Studies 1–2) or Asian Americans (Study 3). The last three items assessed participants' attitudes toward Asian people (Studies 1–2) or Asian Americans (Study 3) on 9-point semantic differential scales (1 = *very bad/very negative/dislike very much*, 9 = *very good/very positive/like very much*). These four items were standardized and averaged together, with higher scores indicating greater positivity toward AAPIs (*α* = 0.89) (see Totton & Rios, 2021, for a similar composite measure).

Finally, participants answered additional demographic questions and provided their best guess as to the race of the person whose picture they had seen (Asian, Black, White, or donť know/donť remember). They were then probed for suspicion and fully debriefed.

## 4 | RESULTS

**FIGURE 1:**
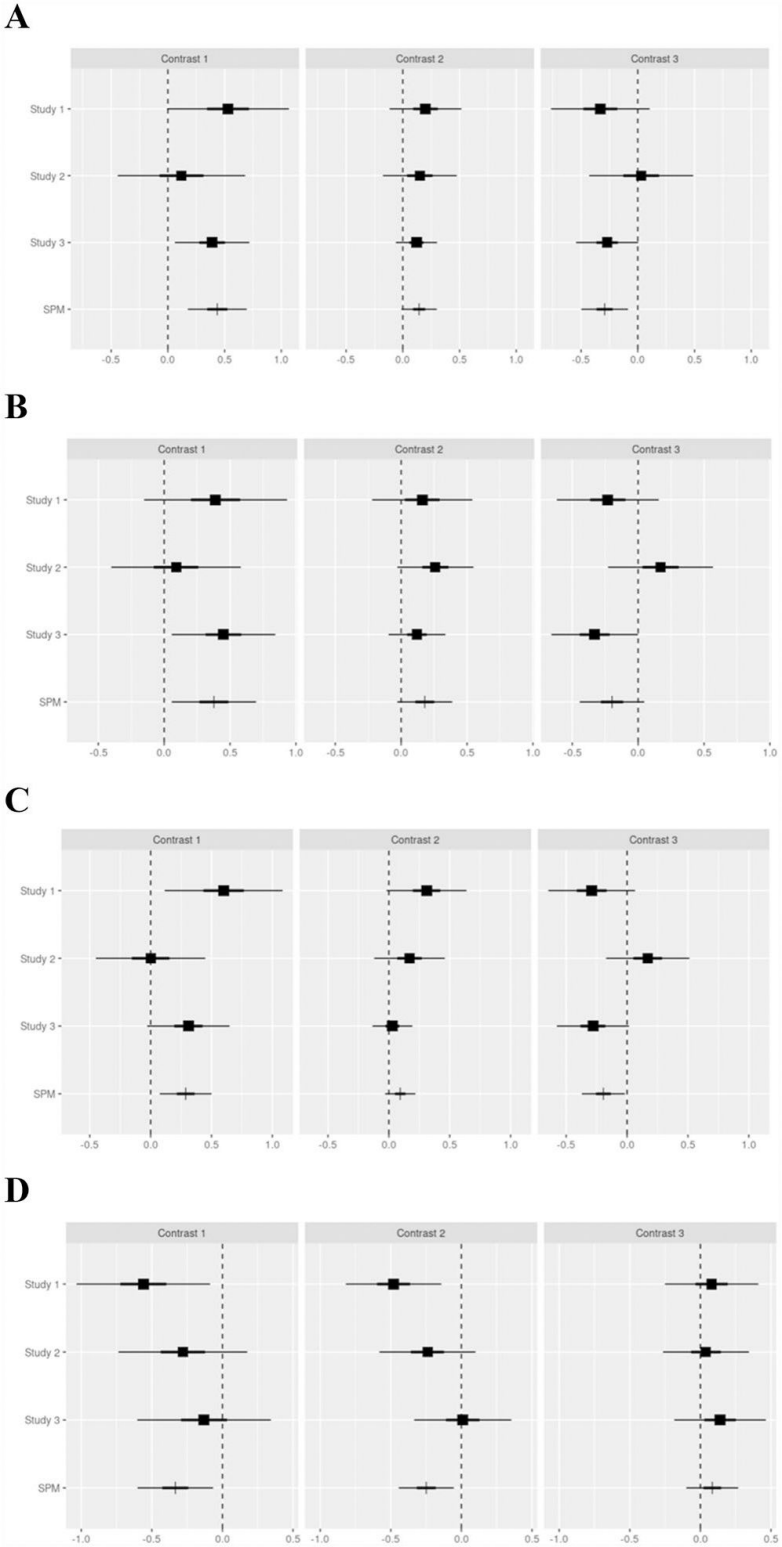
Effect estimates for realistic status threat (a), realistic health threat (b), symbolic threat (c), and positivity toward AAPIs (d). Contrast 1 = two-way interaction; Contrast 2 = AAPI masked versus AAPI unmasked; Contrast 3 = White masked versus White unmasked.

To determine how seeing a masked (vs. unmasked) AAPI individual would affect perceived threat from and positivity toward AAPIs, we used a publicly available ShinyApp (https://blakemcshane.shinyapps.io/spmeta/) to conduct a single-paper meta-analysis (SPM; McShane & Böckenholt, 2017) with two between-participants factors: race condition (AAPI vs. White) and mask condition (masked vs. unmasked). This analysis controlled for differences between the three studies by employing the equivalent of a hierarchical (or multilevel) model fit to the individual-level observations. Table 1 depicts descriptive statistics by cell for perceived threat from and positivity toward AAPIs, and Figure 1 (panels A-D) depicts the single-study and overall estimates of each effect.

Realistic Status Threat. The two-way interaction between race condition and mask condition was significant (*estimate* = 0.37, 95% CI [0.06, 0.68], *p* = 0.021). Planned contrasts revealed that participants who viewed masked AAPI targets perceived nonsignificantly less realistic status threat from AAPIs compared to those who viewed unmasked AAPI targets (*estimate* = 0.18, 95% CI [−0.03,0.40], *p* = 0.101). Conversely, participants who viewed masked White targets perceived nonsignificantly more realistic threat from AAPIs compared to those who viewed unmasked White targets (*estimate* = −0.20, 95% CI [−0.05, 0.45], *p* = 0.112). *I*^2^ was estimated at 55% (95% CI: 5%–78%), suggesting that heterogeneity was medium. In other words, differences between studies beyond those due to the experimental manipulations explained about 55% of the variation in the observations. However, the width of the confidence interval indicates that heterogeneity could range from low to high.

Realistic Health Threat. The two-way interaction between race condition and mask condition was significant (*estimate* = −0.38, 95% CI [−0.58, −0.18], *p* < 0.001. Planned contrasts indicated that participants perceived nonsignificantly less realistic health threat from AAPIs after seeing a masked (vs. unmasked) AAPI target (*estimate* = 0.14, 95% CI [−0.02, 0.30], *p* = 0.070), whereas they perceived significantly more realistic health threat from AAPIs after seeing a masked than unmasked White target (*estimate* = 0.29, 95% CI [0.09, 0.49], *p* = 0.0051). *I*^2^ was estimated at 62% (95% CI: 22%–82%), suggesting that heterogeneity was medium but could range from low to high.

Symbolic Threat. The two-way interaction between race condition and mask condition was significant (*estimate* = −0.31, 95% CI [0.06, 0.68], *p* < 0.001). Planned contrasts revealed that participants who viewed masked AAPI targets perceived nonsignificantly less symbolic threat from AAPIs than did participants who viewed unmasked AAPI targets (*estimate* = 0.09, 95% CI [−0.03, 0.21], *z* = 1.50, *p* = 0.134). Conversely, participants who viewed masked White targets perceived significantly more symbolic threat from AAPIs than did those who viewed unmasked White targets (*estimate* = −0.19, 95% CI [−0.28, −0.10], *p* = 0.035). *I*^2^ was estimated at 26% (95% CI: 0%–66%), suggesting that heterogeneity was low but could range from zero to medium.

Positivity Toward AAPIs. The two-way interaction between race condition and mask condition was significant (*estimate* = 0.23, 95% CI [0.04, 0.42], *p* = 0.015). Planned contrasts indicated that participants reported greater positivity toward AAPIs after seeing a masked than unmasked AAPI target (*estimate* = −0.25, 95% CI [−0.45, −0.05], *p* = 0.012), and equivalent positivity toward AAPIs after seeing masked and unmasked White targets (*estimate* = 0.08, 95% CI [−0.10, 0.26], *p* = 0.368). *I*^2^ was estimated at 68% (95% CI: 36%–84%), suggesting that heterogeneity was medium but could range from low to high.

**FIGURE 2:**
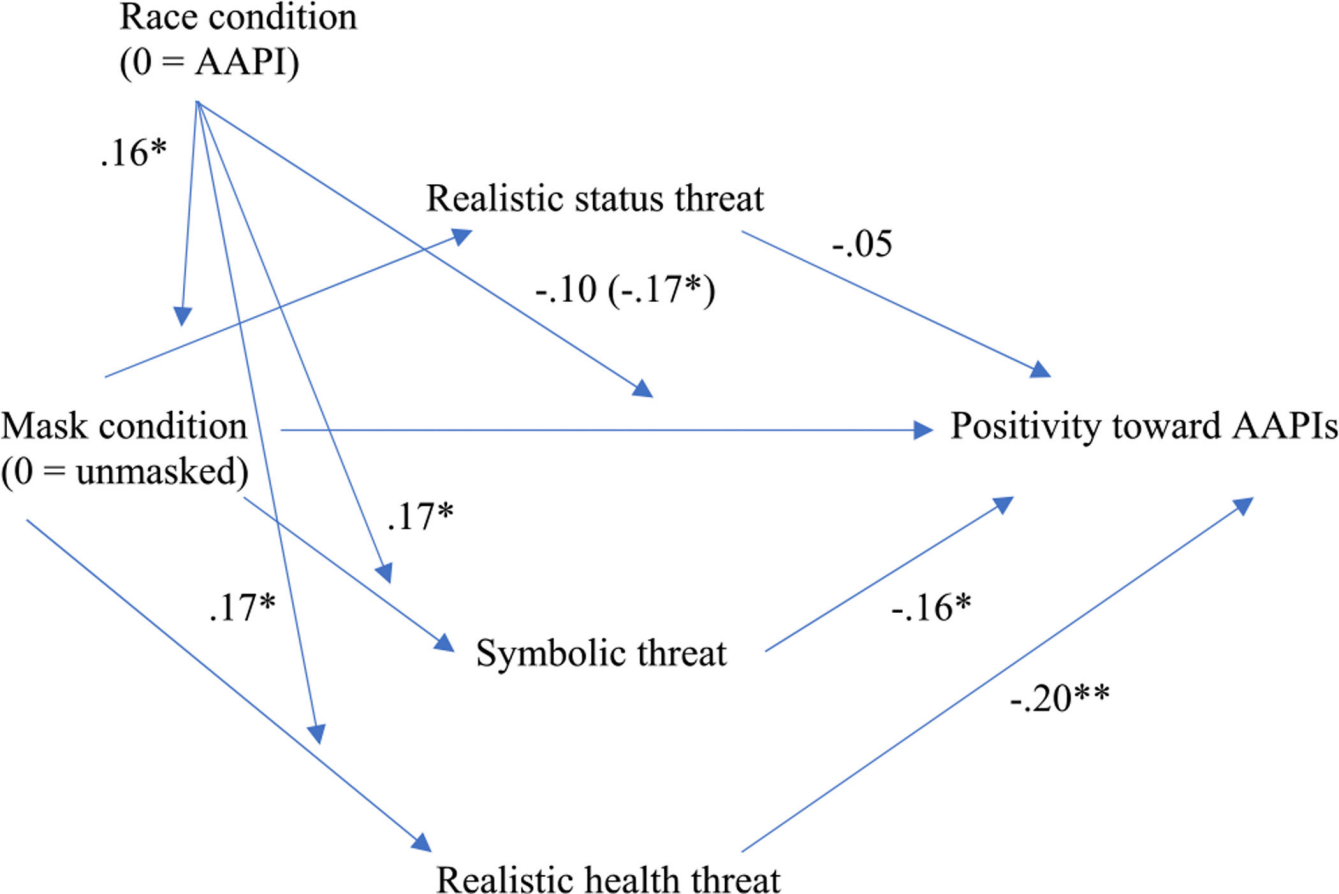
Moderated mediation analysis. *p < 0.05, **p < 0.01.

Moderated Mediation Analysis. To determine whether perceived realistic status threat, perceived symbolic threat, or perceived realistic health threat drove the relationship between mask condition and positivity toward AAPIs among participants exposed to AAPI (but not White) targets, we conducted a moderated mediation analysis with 1000 bootstrap estimates, using PROCESS model 8 (Hayes, 2017). Mask condition was entered as the predictor variable, race condition as the moderator variable, positivity toward AAPIs as the outcome variable, and the three threat types (entered simultaneously) as mediator variables. Study was not included as a factor because it did not significantly interact with race condition or mask condition on any of the mediator or outcome measures. This model is depicted in Figure 2.

The overall index of moderated mediation was significant for perceived symbolic threat (*b* = −0.05, *SE* = 0.03, 95% CI [−0.14, −0.002]) and perceived realistic health threat (*b* = −0.07, *SE* = 0.03, 95% CI [−0.14, −0.01]), but not for perceived realistic status threat (*b* = −0.01, *SE* = 0.02, 95% CI [−0.06, 0.03]). Moreover, for perceived symbolic threat and perceived realistic health threat, the conditional indirect effects of mask condition were significant among participants exposed to AAPI targets (symbolic threat: *b* = 0.03, *SE* = 0.02, 95% CI [0.0003, 0.08]; health threat: *b* = 0.03, *SE* = 0.02, 95% CI [0.0004, 0.08]), but not among participants exposed to White targets (symbolic threat: *b* = −0.02, *SE* = 0.02, 95% CI [−0.08, 0.01]; realistic health threat: *b* = −0.04, *SE* = 0.02, 95% CI [−0.08, 0.01]).

## 5 | DISCUSSION

The COVID-19 pandemic inspired several studies examining perceptions of and attitudes toward mask-wearing (e.g., Howard, 2021; Mackey et al., 2023). Concurrently, there has also been an increase in anti-AAPI attitudes in response to the virus (Dhanani & Franz, 2020; Mandalaywala et al., 2023). However, research examining the potential relationship between mask-wearing and attitudes toward AAPIs is sparse. Across three experiments, we tested whether exposure to masked (vs. unmasked) AAPI individuals can affect White Americans' perceptions of threat from and attitudes toward AAPIs as a group. We began with two competing hypotheses. First, a masked AAPI target might increase negativity toward AAPIs, due to heightened perceptions of realistic threat to the ingroup's health (i.e., seeing AAPIs as possible carriers of COVID-19; Tessler et al., 2020) or symbolic threat to the ingroup's cultural values (i.e., seeing AAPIs as “foreign” and different from the ingroup; Zou & Cheryan, 2017). Second, a masked AAPI target might increase positivity toward AAPIs, due to reduced perceptions of realistic threat to the ingroup's health (i.e., seeing AAPIs as less likely to spread the virus) or symbolic threat (i.e., seeing AAPIs as more American due to compliance with mask mandates).

Collectively, the results of our three experiments supported the second hypothesis. Compared to unmasked AAPI targets, masked AAPI targets increased White Americans' positivity toward AAPIs as a group. Furthermore, among participants exposed to AAPI targets, the effect of mask condition on positivity toward AAPIs was explained in part by reduced perceptions that AAPIs posed a realistic threat to White Americans' physical health and a symbolic threat to White Americans' values. Perceived realistic status threat was not a significant mediator, suggesting that masks primarily function to shift beliefs about AAPIs' likelihood of transmitting COVID-19 (i.e., realistic health threat) and adherence to “American” values surrounding mask-wearing (i.e., symbolic threat). The findings varied somewhat across studies as indicated by the medium heterogeneity estimates for most outcome measures, possibly because the prevalence of movements to combat anti-Asian prejudice (e.g., #StopAsianHate) has fluctuated over time (Cao et al., 2022).

Our findings advance research on the factors contributing to attitudes toward AAPIs during the COVID-19 pandemic. Several studies have demonstrated that anti-AAPI attitudes increased with the onset of COVID-19 (e.g., Dhanani & Franz, 2020; Mandalaywala et al., 2023; Tessler et al., 2020). The present research shows that exposure to AAPIs who are wearing masks can also increase positivity toward AAPIs, via reduced perceptions of threat. Furthermore, our research extends beyond prior studies on how AAPIs believe they will be perceived while wearing masks (Kahn & Money, 2022), by examining how members of these groups are *actually* perceived.

Practically, our results suggest that mask-wearing may sometimes have positive intergroup consequences. Although one prior study (Boykin et al., 2021) found that political conservatives felt less comfortable around AAPIs individuals who were wearing masks, that study did not examine downstream effects of mask-wearing on attitudes toward AAPIs as a group. Encouragingly, we show that seeing a masked AAPI target can ultimately *reduce* perceived threat and hence increase positivity toward AAPIs. An implication of these findings is that in designing public health campaigns and advertisements to promote COVID-safe behaviors, institutions and organizations should make sure people of many backgrounds are represented. For example, a billboard about the importance of wearing masks could include pictures of masked individuals from various racial and ethnic groups. Doing so could help to reduce anti-AAPI attitudes, as could presenting statistics about the high proportion of AAPI healthcare workers.

One limitation of these results is that our samples were, on average, politically liberal. Among more politically conservative populations—or in parts of the US where mask-wearing is less common—our results might be attenuated or even reversed. Another limitation is that our results are generalizable only to White American participants; it remains to be seen whether participants of color would also exhibit reduced threat perceptions from and increased positivity toward AAPIs following exposure to masked AAPI targets. Even so, the present findings suggest that at least when mask-wearing is normative, seeing masked AAPI individuals can mitigate White Americans' anti-AAPI attitudes. Perhaps this also means that as masks become more normalized, AAPIs' fears about how they might be perceived while wearing a mask (Kahn & Money, 2022) may subside as well.

## Supplementary Material

Supplementary materials 1

Supplementary materials 2

SUPPORTING INFORMATION

Additional supporting information can be found online in the Supporting Information section at the end of this article.

## Figures and Tables

**TABLE 1:** Descriptive statistics by cell.

	Realistic status threat	Symbolic threat	Realistic health threat	Positivity
Unmasked	*M* (*SD*), 95% CI	*M* (*SD*), 95% CI	*M* (*SD*), 95% CI	*M* (*SD*), 95% CI
AAPI target	1.38 (0.83), 1.24–1.52	1.28 (0.82), 1.15–1.41	1.33 (0.75), 1.18–1.48	−0.09 (0.88), −0.23–0.06
White target	1.41 (0.78), 1.29–1.55	1.30 (0.81), 1.18–1.42	1.40 (0.96), 1.26–1.53	0.03 (0.87), −0.10–0.16
Masked				
AAPI target	1.20 (0.62), 1.06–1.35	1.12 (0.43), 0.98–1.25	1.17 (0.59), 1.02–1.33	0.15 (0.84), 0.01–0.30
White target	1.58 (1.11), 1.45–1.71	1.45 (0.98), 1.32–1.57	1.60 (1.18), 1.46–1.74	−0.06 (0.87), −0.19–0.08

## Footnotes

1 Perceived realistic status threat from AAPIs increases among US residents reminded of the economic impact of COVID-19 (Dhanani & Franz, 2021). However, because mask-wearing relates more directly to health than to the economy, we do not expect exposure to masked AAPI individuals to affect realistic status threat perceptions.

2 In Studies 2 and 3, we administered conditions in which participants viewed a Black target with (vs. without) a mask. However, given our focus on attitudes toward AAPIs and the difficulties of running a single-paper meta-analysis when not all conditions are the same across studies, we report these results in the Supplementary Materials.
