# Supplementary material for: How does exposure to masked individuals affect White Americans' attitudes toward Asian American and Pacific Islanders?
Source: Soc Personal Psychol Compass. Author manuscript; Available in PMC 2026 Jul 8. (PMC13340608; doi:10.1111/spc3.12819)
Supplement: Supplementary materials 1 [file NIHMS2184112-supplement-Supplementary_materials_1.docx]

**Experimental Stimuli**

*AAPI Female Targets*

**
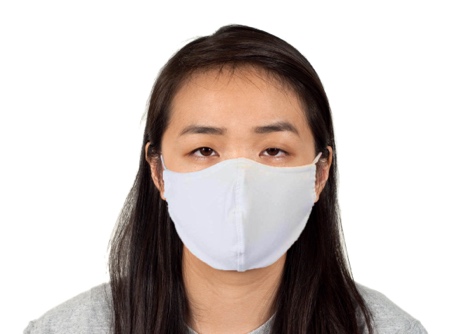

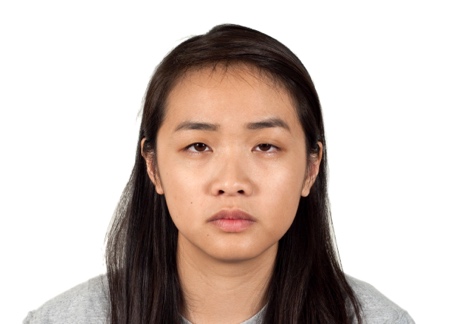
**

*AAPI Male Targets*

**
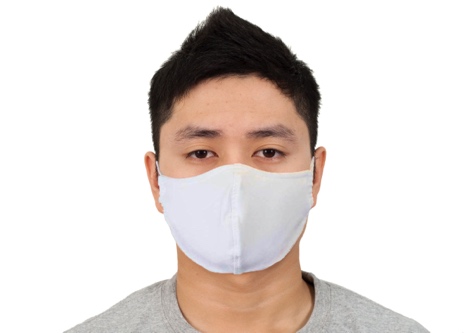

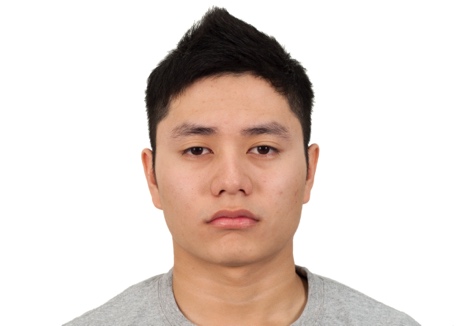
**

*White Female Targets*

**
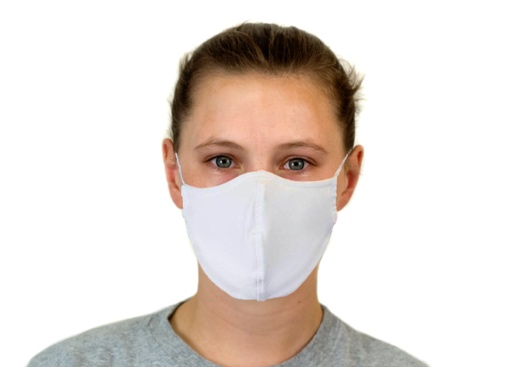

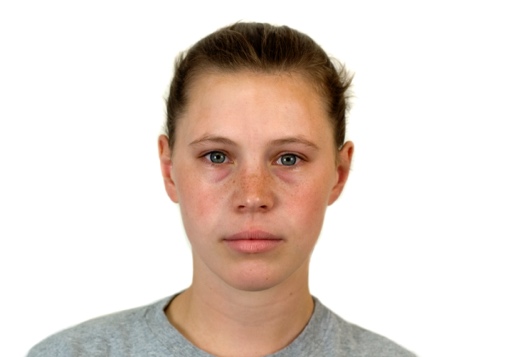
**

*White Male Targets*

**
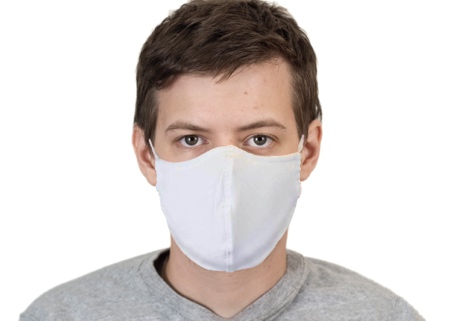

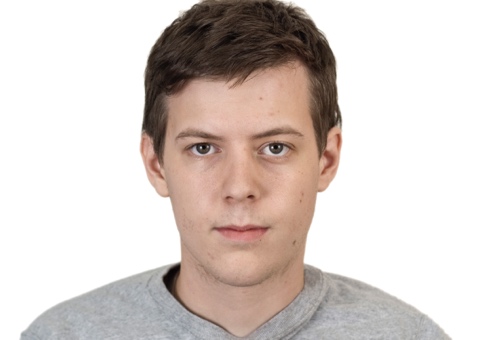
**

*Black Female Targets*

**
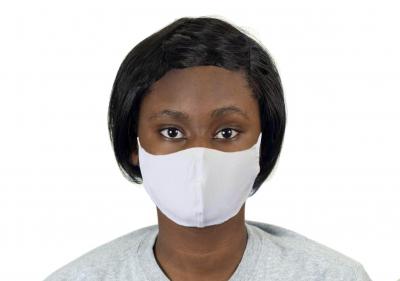

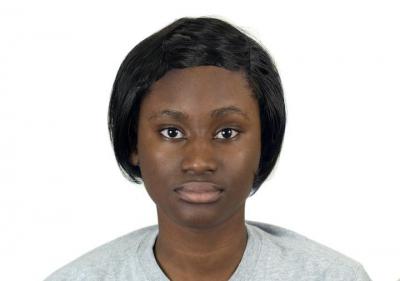
**

*Black Male Targets*

**
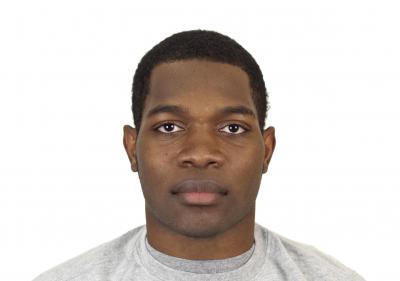

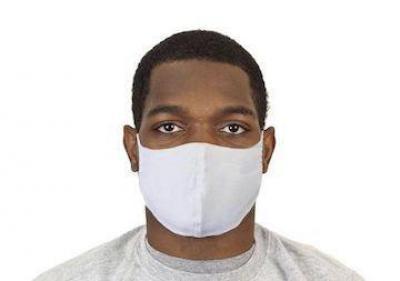
**

**Perceived Threat**

Please answer the following questions about your perceptions of [target group]. *(Not at all, Slightly, Somewhat, Moderately, Quite a bit, Very much, Extremely)*

To what extent do you think that [target group] are threatening other Americans’ power and status? *(realistic status threat)*

To what extent do you think that increases in [target group]’s status will reduce other Americans’ status? *(realistic status threat)*

To what extent do you perceive [target group] as increasing job losses in the US? *(realistic status threat)*

To what extent do you think that [target group] are threatening other Americans’ core values? *(symbolic threat)*

To what extent do you feel that [target group]’s values and practices are negatively affecting American culture? *(symbolic threat)*

To what extent do you feel that [target group]’s values and practices are negatively affecting American values? *(symbolic threat)*

To what extent do you feel that [target group]’s values and practices are negatively affecting other Americans’ way of life? *(symbolic threat)*

To what extent do you think that [target group] are threatening the health of other groups in the US? *(realistic health threat)*

To what extent do you think that [target group] is threatening other Americans’ physical safety? *(realistic health threat)*

To what extent do you perceive [target group] as increasing the prevalence of diseases in the US? *(realistic health threat)*

To what extent do you feel that [target group] are negatively affecting other Americans’ overall health? *(realistic health threat)*

**Study 1**

**Method**

**Participants.** Two hundred and fifty-two White US residents completed this study on Prolific Academic, a crowdsourcing platform that allows for selection of participants based on demographic characteristics, in exchange for $1.00. The data from three participants who did not self-identify as White, 37 participants who did not correctly identify the race of the target person, and one participant who took more than an hour to complete the study were omitted from analyses. The final sample thus consisted of 211 individuals (130 women, 78 men, 1 transgender woman, 2 gender non-binary/gender-fluid; *M*_age_ = 34.44, *SD =* 12.91).

Participants were randomly assigned to view a picture of an Asian American/Pacific Islander (AAPI; *n* = 92) or White (*n* = 119) individual, who was either masked (*n* = 102) or unmasked (*n* = 109).

**Procedure and Materials.** Data were collected in May 2020. After providing informed consent, participants read that the purpose of the study was to examine perceptions of different individuals and groups. They then answered a few basic demographic questions. On the following screen, participants viewed a photograph of their assigned target person. As described above, the target person was either AAPI or White and was either wearing a plain white cloth mask over their nose and mouth (photoshopped onto the picture) or was not wearing a mask. The gender of the target person always matched the gender of the participant; participants who self-identified as gender non-binary/gender-fluid were randomly assigned to view either a male or female target. The photographs were taken from the Chicago Face Database (Ma et al., 2015), which contains data on independent raters’ perceptions of each target person’s race, gender, age, attractiveness, and other characteristics (e.g., emotional expressions). For the present study, all selected target persons were rated as being in their early- to mid-20s and of roughly average physical attractiveness (i.e., just above a 3 out of 5); in addition, raters needed to correctly identify the target’s race at least 95% of the time.

Participants were asked to imagine that they had seen the target person at their local grocery store. Below the photograph, they responded to several filler items, unrelated to the present research question, about their perceptions of the target person to disguise the purpose of the study.

Participants then completed an 11-item measure of perceived threat from AAPIs. This measure contained three items assessing perceived realistic threat to group status and resources (e.g., “To what extent do you think that increases in Asian people’s status will reduce other Americans’ status?”; *M* = 1.46, *SD* = .95, α = .92), four items assessing perceived symbolic threat to group values (e.g., “To what extent do you feel that Asian people are threatening other Americans’ core values?”; *M* = 1.31, *SD* = .85, α = .95), and four items assessing perceived realistic threat to group health (e.g., “To what extent do you think that Asian people are threatening the health of other groups in the US?”; *M* = 1.44, *SD* = .99, α = .97).

Next, using “feeling thermometer” scales from 0 (*no warmth at all*) to 100 (*complete warmth*), participants indicated their feelings of warmth toward AAPIs, worded in this study as “Asian people” (*M* = 71.03, *SD* = 23.10). Participants also completed three items assessing their attitudes toward AAPIs (“Asian people”) on 9-point semantic differential scales (1 = *very bad/very negative/dislike very much*, 9 = *very good/very positive/like very much*; *M =* 6.88, *SD =* 1.55, α = .91). These three items and the “feeling thermometer” measure were standardized and averaged together, with higher scores indicating greater positivity toward AAPIs (α = .88).

Finally, participants answered additional demographic questions (i.e., that were not administered at the beginning of the study) and provided their best guess as to the race of the person whose picture they had seen (Asian, Black, White, or don’t know/don’t remember). They were then probed for suspicion and fully debriefed.

**Results**

We predicted that the differences between the masked and unmasked conditions in perceived threat from and positivity toward AAPIs would be most pronounced when the target person was AAPI (versus White). However, we did not have strong predictions about the direction of the effects. That is, we anticipated that seeing an AAPI wearing a mask could elicit either greater positivity (if the mask mitigates threat perceptions) or greater negativity (if the mask *exacerbates* threat perceptions) toward AAPIs as a whole, compared to seeing an AAPI not wearing a mask. We conducted 2 (race condition: AAPI vs. White) x 2 (mask condition: masked vs. unmasked) between-participants ANOVAs on each threat measure, as well as the measure of positivity toward AAPIs.

**Perceived Threat.** On perceived realistic status threat, there were no main effects of race condition or mask condition (*p*s > .148), nor did the two-way interaction reach significance (*p =* .174).

On perceived symbolic threat, no main effects emerged (*p*s > .333), but the two-way interaction was significant, *F*(1, 207) = 5.51, *p =* .020, η^2^_ρ_ = .03. Simple effects tests revealed that participants who viewed the unmasked (versus masked) AAPI perceived nonsignificantly greater symbolic threat from AAPIs, *F*(1, 207) = 3.22, *p* = .074, η^2^_ρ_ = .02, but participants who viewed the unmasked (versus masked) White target person perceived significantly *less* symbolic threat from AAPIs, *F*(1, 207) = 6.43, *p* = .012, η^2^_ρ_ = .03. Additionally, participants in the masked condition perceived lower symbolic threat from AAPI when the target person was AAPI (*M* = 1.11, *SD =* .47, 95% CI [.83, 1.39]) than White (*M =* 1.54, *SD =* 1.09, 95% CI [1.30, 1.78]), *F*(1, 207) = 6.53, *p =* .011, η^2^_ρ_ = .03, whereas participants in the unmasked condition did not differ in perceived symbolic threat from AAPIs based on whether the target person was AAPI (*M* = 1.42, *SD =* 1.06, 95% CI [1.16, 1.69]) or White (*M =* 1.25, *SD =* .88, 95% CI [1.01, 1.48]), *F*(1, 207) = .99, *p =* .321, η^2^_ρ_ = .01.

On perceived realistic health threat, there was no main effect of mask condition (*p* = .647), but there was a main effect of race condition, such that participants perceived less realistic health threat from AAPIs after viewing an AAPI target (*M* = 1.19, *SD* = .60, 95% CI [1.08, 1.51]) than a White target (*M* = 1.76, *SD* = 1.37, 95% CI [1.41, 1.79]). The two-way interaction did not reach conventional levels of significance, *F*(1, 207) = 3.37, *p* = .068, η^2^_ρ_ = .02. Decomposition of the interaction revealed that the comparisons between masked and unmasked AAPI targets, as well as between masked and unmasked White targets, were not significant (*p*s > .084). However, that in the masked condition, participants perceived less heath threat from AAPIs when the target person was AAPI (*M* = 1.20, *SD* = .60, 95% CI [.88, 1.51]) than White (*M* = 1.76, *SD* = 1.37, 95% CI [1.49, 2.03]), *F*(1, 207) = 7.25, *p =* .008, η^2^_ρ_ = .03. In the unmasked condition, perceived health threat from AAPIs did not differ by whether the target person was AAPI (*M* = 1.40, *SD* = .93, 95% CI [1.10, 1.69]) or White (*M* = 1.43, *SD* = 1.02, 95% CI [1.16, 1.70]), *F*(1, 207) = .02, *p* = .877, η^2^_ρ_  < .001.

**Positivity Toward AAPIs.** There were no main effects (*p*s > .105), but the interaction between race condition and mask condition was significant, *F*(1, 207) = 5.18, *p* = .024, η^2^_ρ_ = .02. According to simple effects tests, participants exhibited greater positivity toward AAPIs after viewing a masked than unmasked AAPI target, *F*(1, 207) = 6.74, *p* = .010, η^2^_ρ_ = .03, but participants did not differ in positivity toward AAPIs based on whether they viewed a masked or unmasked White target person, *F*(1, 207) = .242, *p* = .523, η^2^_ρ_ = .001. Additionally, participants in the masked condition exhibited greater positivity toward AAPIs when the target person was AAPI (*M* = .16, *SD* = .70, 95% CI [-.10, .43]) than White (*M* = -.21, *SD* = .93, 95% CI [-.43, .02]), *F*(1, 207) = 4.42, *p* = .037, η^2^_ρ_ = .02. By contrast, participants in the unmasked condition exhibited statistically equivalent positivity toward AAPIs whether the target person was AAPI (*M* = -.32, *SD* = .95, 95% CI [-.56, -.07]) or White (*M* = -.13, *SD* = .91, 95% CI [-.35, .10]), *F*(1, 207) = 1.21, *p* = .274, η^2^_ρ_ = .006.

**Study 2**

**Method**

**Participants.** Three hundred and fifty-nine university students participated in exchange for partial course credit. The data from 57 participants who self-identified as non-White, 25 participants who did not correctly indicate the race of the target person, and 5 participants who took more than an hour to complete the study were omitted, leaving 272 individuals in the final sample (206 women, 60 men, 1 transgender woman, 2 transgender men, 2 gender non-binary/gender-fluid, 1 another gender identity; *M*_age_ = 19.36, *SD* = 3.63). Participants were randomly assigned to view a picture of an Asian (*n* = 85), Black (*n =* 83), or White (*n =* 104) individual, who was either masked (*n* = 135) or unmasked (*n =* 137).

**Procedure and Materials.** Data were collected between September and December 2020. The procedure was similar to that of Study 1, with three modifications. First, we added a condition in which the target person was Black so that we could test whether exposure to a masked person from a non-AAPI racial/ethnic minority group would also increase positivity toward members of that group. Second, because wearing masks in grocery stores was required in most US states by the time this study was run, participants were instead asked to imagine that they had seen the target person in a local park. Third, participants in the Black target person conditions answered the 11 questions about perceived threat from, and the four questions about positivity toward, Black people. (These questions were the same as the questions about Asian people in Study 1, only they were worded to pertain to Black people.) Participants in the AAPI target person conditions answered the corresponding questions about AAPIs, whereas participants in the White target person conditions answered the questions about both AAPIs and Black people.

**Results**

Due to the study design (i.e., participants in the Black target person conditions did not see the questions about AAPIs and vice versa), the results were analyzed using two separate ANOVAs: 2 (race condition: AAPI vs. White) x 2 (mask condition: masked vs. unmasked), and 2 (race condition (Black vs. White) x 2 (mask condition: masked vs. unmasked). Tables S1 and S2 depict descriptive statistics by cell for perceived threat from and positivity toward AAPIs (Table S1) and Black individuals (Table S2).

**Perceived Threat from AAPIs.** On perceived realistic threat to group status, participants perceived less threat from AAPIs after viewing an AAPI target (*M* = 1.28, *SD* = .68, 95% CI [1.09, 1.47]) than a White target (*M* = 1.64, *SD* = 1.03, 95% CI [1.47, 1.81]), *F*(1, 185) = 7.29, *p* = .008, η^2^_ρ_ = .04. There was no main effect of mask condition (*p* = .101), nor was there a two-way interaction, *F*(1, 185) = .11, *p* = .746, η^2^_ρ_ = .001.

On perceived symbolic threat, participants perceived less threat from AAPIs after viewing an AAPI target (*M* = 1.20, *SD* = .68, 95% CI [1.03, 1.38]) than a White target(*M* = 1.44, *SD* = .89, 95% CI [1.29, 1.60]), *F*(1, 185) = 4.17, *p* = .043, η^2^_ρ_ = .02. There was no main effect of mask condition (*p* = .161), nor was there a two-way interaction, *F*(1, 185) < .001, *p* = .984, η^2^_ρ_ < .001.

On perceived realistic threat to group health, participants perceived less threat from AAPIs after viewing an AAPI target (*M* = 1.30, *SD* = .76, 95% CI [1.09, 1.52]) than a White target (*M* = 1.65, *SD* = 1.18, 95% CI [1.46, 1.85]), *F*(1, 185) = 5.55, *p* = .019, η^2^_ρ_ = .03. No other effects were significant (*F*s < 1, *p*s > .531).

**Positivity Toward AAPIs.** There were no main effects of mask condition or race condition (*p*s > .393), nor was there a two-way interaction, *F*(1, 185) = 1.38, *p* = .242, η^2^_ρ_ = .007.

**Perceived Threat from Black individuals.** Neither main effect reached significance (*p*s > .054), nor did the two-way interaction, *F*(1, 185) = .93, *p* = .337, η^2^_ρ_ = .005.

On perceived symbolic threat, participants perceived less threat from Black individuals after viewing a Black target (*M* = 1.24, *SD* = .64, 95% CI [1.06, 1.42]), than a White target, (*M* = 1.51, *SD* = .96, 95% CI [1.34, 1.67]), *F*(1, 185) = 4.56, *p* = .034, η^2^_ρ_ = .02. There was no main effect of mask condition (*p* = .442), nor was there a two-way interaction, *F*(1, 185) = 2.25, *p* = .135, η^2^_ρ_ = .01.

On perceived realistic threat to group health, there were no main effects (*p*s > .171), nor was there a two-way interaction, *F*(1, 185) = 2.00, *p* = .159, η^2^_ρ_ = .01.

**Positivity Toward Black individuals.** Overall, participants felt more positively toward Black individuals after viewing a Black target (*M* = .20, *SD* = .87, 95% CI [-.005, .40]) than a White target (*M* = -.15, *SD* = .96, 95% CI [-.33, .03]), *F*(1, 185) = 6.45, *p* = .012, η^2^_ρ_ = .03. There was no main effect of mask condition (*p* = .587), nor was there a two-way interaction, *F*(1, 185) = .18, *p* = .676, η^2^_ρ_ = .001.

**Study 3**

**Method**

**Participants.** Three hundred and fifty White US residents participated in exchange for $1.00. The data from five participants who did not self-identify as White and 35 participants who did not correctly identify the race of the target person were omitted from analyses. The final sample thus consisted of 309 individuals (159 women, 134 men, 4 transgender women, 2 transgender men, 9 gender non-binary/gender-fluid, 1 another gender identity; *M*_age_ = 36.61, *SD =* 13.88). These individuals were randomly assigned to view a picture of an Asian (*n* = 102), Black (*n* = 95), or White (*n* = 112) target person, who was either masked (*n* = 154) or unmasked (*n* = 155).

**Procedure and Materials.** The procedure was identical to that of Study 2, with two modifications. First, the setting was changed from a local park to “a fairly busy local street” to both increase generalizability and increase the potential stakes associated with encountering an unmasked person. Second, participants in all three race conditions answered the questions about perceived threat from and positivity (the three semantic differential items plus the “feeling thermometer” item from Study 1) toward both AAPIs and Black Americans. As in Study 1, the four positivity items were averaged together due to the high correlations between the semantic differential measure and the feeling thermometer item (*r*s > .62, *p*s < .001). We modified the wording from “Asian people/Black people” to “Asian/Black Americans” to avoid implying that these groups were un-American (Cheryan & Monin, 2005).

**Results and Discussion**

The results on all dependent measures were analyzed using 3 (race condition: AAPI, Black, White) x 3 (mask condition: masked vs. unmasked) between-participants ANOVAs. See Table S3 for descriptive statistics for each measure by cell.

**Perceived Threat from AAPIs.** There were no main effects of race condition or mask condition on any of the three perceived threat measures (*p*s > .141). The only two-way interaction to emerge was on perceived realistic threat to group status, *F*(1, 303) = 3.22, *p* = .041, η^2^_ρ_ = .02. Simple main effects tests indicated that participants perceived statistically equivalent group status threat from AAPIs after viewing masked and unmasked AAPI targets, *F*(1, 303) = .66, *p* = .418, η^2^_ρ_ = .002, and Black targets, *F*(1, 304) = .89, *p* = .347, η^2^_ρ_ = .003. However, participants perceived more group status threat from AAPIs after viewing a masked than unmasked White target, *F*(1, 303) = 5.03, *p* = .026, η^2^_ρ_ = .02. No other simple main effects were significant (*p*s > .086).

**Positivity Toward AAPIs.** There were no main effects of race condition or mask condition, nor was there a two-way interaction (*p*s > .584).

**Perceived Threat from Black Americans.** There were no main effects of race condition or mask condition on any of the three perceived threat measures (*p*s > .328). The only two-way interaction to emerge was on perceived realistic threat to group health, *F*(1, 303) = 3.23, *p* = .041, η^2^_ρ_ = .02. Though none of the simple main effect tests were significant, the pattern of the interaction suggested that participants perceived less health realistic threat from Black Americans after viewing a masked (versus unmasked) Black or AAPI target, whereas they perceived *more* realistic health threat from Black Americans after viewing a masked (versus unmasked) White target.

**Positivity Toward Black Americans.** There were no main effects of race condition or mask condition, nor was there a two-way interaction (*p*s > .498).

**Table S1**

|  | Realistic status threat | Symbolic threat | Realistic health threat | Positivity |
| --- | --- | --- | --- | --- |
| Unmasked | *M* (*SD*), CI | *M* (*SD*), CI | *M* (*SD*), CI | *M* (*SD*), CI |
| AAPI target | 1.41 (.80),  1.14-1.68 | 1.29 (.89),  1.04-1.53 | 1.38 (.77),  1.07-1.69 | .17 (.81),  -.07-.42 |
| White target | 1.72 (1.07),  1.48-1.96 | 1.53 (.99),  1.31-1.75 | 1.67 (1.26),  1.39-1.95 | .21 (.84),  -.01-.43 |

Masked

| AAPI target | 1.15 (.53),  .89-1.42 | 1.12 (.37),  .88-1.37 | 1.23 (.76),  .92-1.53 | .41 (.79),  .17-.65 |
| --- | --- | --- | --- | --- |
| White target | 1.55 (1.00),  1.31-1.80 | 1.36 (.78),  1.14-1.58 | 1.64 (1.12),  1.36-1.92 | .17 (.74),  -.05-.39 |

**Table S2**

|  | Realistic status threat | Symbolic threat | Realistic health threat | Positivity |
| --- | --- | --- | --- | --- |
| Unmasked | *M* (*SD*), CI | *M* (*SD*), CI | *M* (*SD*), CI | *M* (*SD*), CI |
| Black target | 1.35 (.62),  1.08-1.62 | 1.20 (.51),  .95-1.45 | 1.20 (.52),  1.00-1.40 | .20 (.90),  -.08-.48 |
| White target | 1.73 (1.30),  1.49-1.98 | 1.64 (1.09),  1.42-1.87 | 1.47 (.93),  1.29-1.65 | -.09 (.99),  -.34-.17 |

Masked

| Black target | 1.25 (.53),  .97-1.53 | 1.29 (.76),  1.03-1.55 | 1.22 (.52),  1.02-1.43 | .19 (.83),  -.08-.48 |
| --- | --- | --- | --- | --- |
| White target | 1.38 (.80),  1.13-1.62 | 1.37 (.81),  1.14-1.59 | 1.23 (.55),  1.04-1.40 | -.22 (.94),  -.47-.04 |

**Table S3**

|  | Realistic status threat | Symbolic threat | Realistic health threat | Positivity |
| --- | --- | --- | --- | --- |
| Unmasked | *M* (*SD*), CI | *M* (*SD*), CI | *M* (*SD*), CI | *M* (*SD*), CI |
| AAPI target | 1.28 (.56),  1.08-1.49 | 1.15 (.40),  .96-1.35 | 1.22 (.54),  1.04-1.40 | -.08 (.82),  -.31-.16 |
| White target | 1.16 (.48),  .96-1.37 | 1.15 (.45),  .97-1.34 | 1.11 (.31),  .93-1.28 | .03 (.85),  -.20-.26 |
| Black target | 1.42 (.98),  1.20-1.65 | 1.37 (.92),  1.16-1.58 | 1.31 (.74),  1.11-1.51 | -.11 (.88),  -.37-.15 |

Masked

| AAPI target | 1.16 (.55),  .94-1.38 | 1.12 (.44),  .92-1.33 | 1.10 (.39),  .91-1.29 | -.08 (.94),  -.33-.17 |
| --- | --- | --- | --- | --- |
| White target | 1.49 (1.14),  1.29-1.70 | 1.43 (1.03),  1.24-1.62 | 1.38 (.99),  1.20-1.56 | -.11 (.90),  -.34-.13 |
| Black target | 1.27 (.71),  1.06-1.49 | 1.28 (.84),  1.09-1.48 | 1.26 (.82),  1.08-1.45 | -.14 (.87),  -.38-.11 |
