# Supplementary material for: How does exposure to masked individuals affect White Americans' attitudes toward Asian American and Pacific Islanders?
Source: Soc Personal Psychol Compass. Author manuscript; Available in PMC 2026 Jul 8. (PMC13340608; doi:10.1111/spc3.12819)
Supplement: Supplementary materials 2 [file NIHMS2184112-supplement-Supplementary_materials_2.docx]

Descriptive Statistics by Cell, Study 1 (Table S1),

Descriptive Statistics by Cell, Study 2 (Table S2),

Descriptive Statistics by Cell, Study 3 (Table S3).
